# The impact of cotrimoxazole in idiopathic granulomatous mastitis treatment

**DOI:** 10.1016/j.ijscr.2024.109959

**Published:** 2024-06-27

**Authors:** Majid Samsami, Fatemeh Parsaeian, Alireza Haghbin Toutounchi, Hojatolah Khoshnoudi, Hamed Tahmasbi

**Affiliations:** Department of General Surgery, Imam Hossein medical and educational center, Shahid Beheshti University of medical sciences, Tehran, Iran

**Keywords:** Breast, Idiopathic granulomatous mastitis, Mastitis, Trimethoprim, Sulfamethoxazole drug combination

## Abstract

**Introduction and importance:**

Idiopathic granulomatous mastitis (IGM) is a benign inflammatory breast disease, commonly presented with a sensitive breast lump and developing scars. Currently, there is no definitive treatment for IGM but Antibiotics, steroids, immunosuppressive drugs or a surgical treatments are the usual options. This case series aimed to evaluate the effectiveness of cotrimoxazole in treatment of IGM as there is no clinical consensus on the best and most widely acknowledged therapeutic management for IGM.

**Case presentation:**

All IGM patients were treated by Cotrimoxazole (800 mg BD for one week), and they were assessed at a month, 3 months, and 6 months after that. The primary outcome was an improvement in presenting complaints and symptoms such as palpable mass, bulging, pain, erythema and hypersensitivity of breast skin, breast discharge and fluctuation. The secondary outcome was the refractory rate within 6 months. Number of 20 patients were included. At the baseline, participants exhibited various symptoms such as bulging, pain and erythema (100 %), breast discharge (80 %), and fluctuation (30 %). After the intervention, there was a significant decrease in the prevalence of symptoms over the study period. The prevalence of bulging and pain, erythema, discharge, and fluctuation symptoms were decreasedto 5 %, 0 %, and 0 %, respectively. The refractory rate of IGM within six months of cotrimoxazole treatment was estimated 30 %.

**Clinical discussion:**

In this study, the treatment approach did not involve corticosteroids and invasive procedures and the recurrence rate of IGM within the six months was lower than in similar studies that employed steroids alone or any more invasive treatments. Additionally, our study showed a high healing rate with resolution of inflammation, pain, discharge, and fluctuation. These results suggest that cotrimoxazole may be a more favorable option than high-dose corticosteroids and a comparable alternative to low-dose corticosteroids regarding recurrence rates.

**Conclusion:**

Cotrimoxazole may be an effective treatment option for idiopathic granulomatous mastitis. However, further research is needed on different treatment options.

## Highlights

• The results suggest that cotrimoxazole treatment maybe effective in reducing symptoms associated with IGM.
• The prevalence of breast discharge, pain, erythema, and fluctuation all decreased significantly after the intervention.
• We estimated the recurrence rate of IGM within the six months of cotrimoxazole treatment to be 30 % during six months follow-up.

## 1 Introduction

Kessler and Wolloch first reported idiopathic granulomatous mastitis (IGM) as an uncommon and benign inflammatory illness of the breast in 1972 [1]. Chronic necrotic granulomas, an inflammation of the lobules, define IGM [2]. Women of reproductive age are more likely to develop idiopathic granulomatous mastitis [3,4]. The most typical phase is between 2 and 6 years after childbirth [5]. This condition is more common among Asian, Hispanic, and Middle Eastern people and is frequently related to breastfeeding or hyperprolactinemia [6,7].

Although the exact origin of IGM has not been discovered yet, research has linked factors such as breastfeeding, reaction to trauma, immunological and connective tissue diseases, hormonal imbalance, oral contraceptives, α1-antitrypsin deficiency, and smoking [9,10]. There are also further theories about the disease's connection to infectious agents such as TB, actinomycosis, blastomycosis, and Corynebacterium species or proliferative diseases like sarcoidosis [11,12]. Studies indicate that the most common symptoms of this condition are one-sided, uncomfortable, swelling breast mass with redness or skin ulcer, drainage from the duct and nipple, and nipple indentation, and it can also lead to a fistula, sinus tract, and scar development [9,13,14]. While unilateral clinical presentation is more common, bilateral IGM has been described in some cases [10].

Currently, there is no an approved definitive treatment for IGM. Antibiotic therapy, steroid therapy, and immunosuppressive drugs or an invasive technique such as surgery or abscess drainage are the possible options [15]. The surgical approach has been controversial because of poor wound healing, fistula formation, and recurrence [16]. Physicians commonly use antibiotics and corticosteroids [17]. However, there is no clinical consensus on the best and most widely acknowledged therapeutic management for IGM. Thus, we decided to conduct a study in order to reveal the impact of cotrimoxazole in treating IGM in patients referred to the university medical center during 2021–2023.

## 2 Materials and methods

### 2.1 Study design

The main goal of this case series is to evaluate the impact of Cotrimoxazole as a single therapy in IGM patients. The participants' outcomes are assessed at the baseline and at the certain followup period, and the results are examined for ascertaining the treatment's effectiveness. This study was conducted after registration in the ethical committee of University of Medical Sciences. All attendees were given written inform consent. The work has been reported in line with the PROCESS criteria [29].

### 2.2 Data collection

The inclusion criteria for the study participants included new IGM patients diagnosed by pathology and referred during 2021–2023. The exclusion criteria included any diagnosis other than IGM, patients with no definite pathological diagnosis as IGM, patients treated for IGM before this setting, incomplete medical records or insufficient follow-up data, patients with a history of allergy or side effects to cotrimoxazole, underlying medical conditions that may interfere with the evaluation of the effect of cotrimoxazole on idiopathic granulomatous mastitis, corticosteroid consumption in the last three months, history of taking contraceptive pills, history of hormone therapy and smokers as a confounding factor. All IGM patients were treated by Cotrimoxazole (800 mg BD for one week), and they were assessed at a month, 3 months, and 6 months after that. Aspiration is performed if the collection has reached the skin's surface after a week, and the antibiotic may be continued depending on the degree of skin erythema. However, if the symptoms improve, we discontinue the antibiotics. The primary outcome assessed in this study were improvement in presenting complaints and symptoms including palpable mass, bulging, pain, erythema and hypersensitivity of breast skin, breast discharge and fluctuation. The secondary outcome was the recurrence rate during the follow-up period. All patients are assessed after a month, 3 months, and 6 months.

### 2.3 Statistical analysis

Descriptive statistics were calculated to summarize the characteristics of the study participants, including demographic information, clinical features, and baseline measurements. Multiple regression analysis was conducted to assess the effectiveness of cotrimoxazole in the treatment of IGM and to explore potential predictors of treatment response. The primary outcomes were considered dependent variables, while potential predictors (age, history of breastfeeding, baseline symptoms) were included as independent variables. A *p*-value <0.05 was considered significant.

## 3 Results

The study included 20 participants who met the inclusion criteria. The mean age of the study participants was 31.45 ± 8.46 years. Among the patients included, 80 % had a history of breastfeeding, while 20 % did not mention their breastfeeding history.

Baseline information, including signs and symptoms, was gathered. Complaint of palpable mass, pain and erythema and hypersensitivity of breast skin were present in 100 % of patients. Breast discharge was observed in 80 % of the participants. Fluctuation was observed in 30 % of the participants [Table 1].

**Table 1:** Changes in the prevalence of main sign and symptoms of idiopathic granulomatous mastitis

	Time				p-value
	Baseline	1 month	3 months	6 months	
Bulging and Pain	20 (100 %)	2 (10 %)	4 (20 %)	1 (5 %)	<0.001
Erythema and skin sensitivity	20 (100 %)	2 (10 %)	4 (20 %)	1 (5 %)	<0.001
Breast discharge	5 (25.0 %)	2 (10 %)	0 (0 %)	0 (0 %)	0.011
Fluctuation	6 (30 %)	1 (5 %)	0 (0 %)	0 (0 %)	0.001

**Fig. 1:**
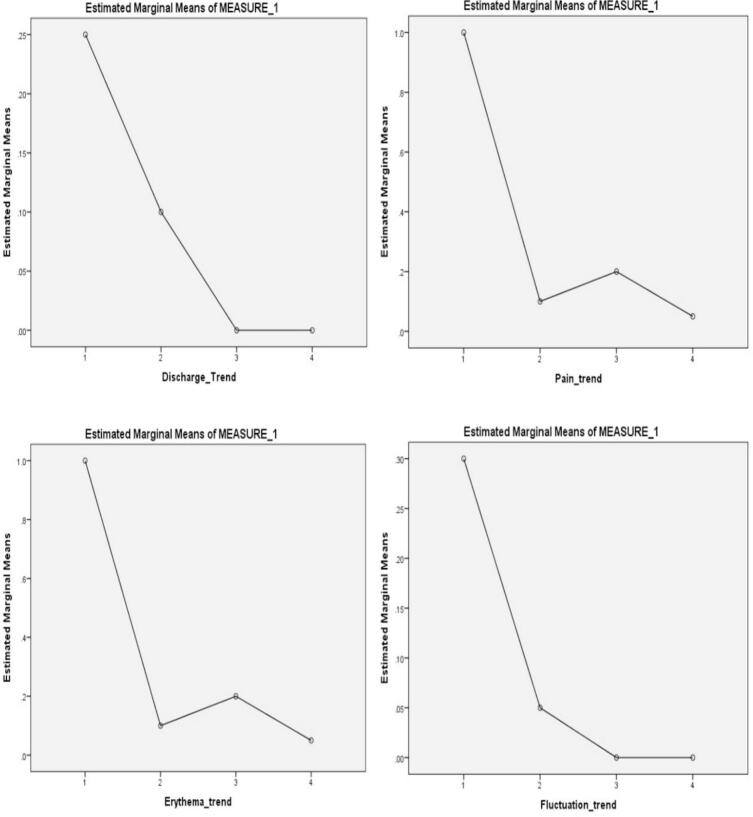
Trend analysis of primary outcomes of the study. Corresponding p-value for observed trend is 0.011, < 0.001, <0.001, and 0.001 for breast discharge, pain, erythema, and fluctuation.

Table 1 depicts the differences in the prevalence of the mentioned signs and symptoms after administration of cotrimoxazole. The prevalence of most breast discharge symptoms decreased from 25 % before the intervention to 10 %, 0 %, and 0 % at one month, three months, and six months, respectively. This decreasing trend in the prevalence of discharge symptoms was statistically significant (*p*-value = 0.011). All patients experienced bulging and pain at first. However, during the follow-up periods, the prevalence of pain decreased to 10 %, 20 %, and 5 % at one month, three months, and six months, respectively. The decrease in the prevalence of bulging and pain was statistically significant (*p*-value <0.001). At the start of the study, all patients exhibited erythema. Over one, three, and six months after the intervention, the prevalence of erythema decreased to 10 %, 20 %, and 5 %, respectively. These changes in erythema prevalence were statistically significant (*p*-value <0.001). Initially, 30 % of the participants had fluctuation symptoms. After one month of the intervention, the prevalence decreased to 5 %, and at three and six months, it reached 0 %. The decreasing trend in the prevalence of fluctuation symptoms throughout the study was statistically significant (*p*-value = 0.001). Fig. 1 illustrates the trends of the primary outcomes of our study [Fig. 1]. Finally, the recurrence rate of IGM was estimated to be 30 % during the six-month follow-up.

## 4 Discussion

The current study aimed to evaluate the effectiveness of cotrimoxazole in the management of IGM. It is essential to rule out malignancy and other potential causes of mastitis before diagnosing IGM. Histopathological examination is often necessary for a definitive diagnosis, due to the high rate of misdiagnosis between IGM and breast cancer [18]. In a large-scale cohort study conducted in Sweden, no association was found between breast cancer and inflammatory breast disease, including IGM [19]. There is currently no evidence to suggest that IGM or other types of mastitis are risk factors for breast cancer [20]. Notably, we excluded all patients with possible breast cancer diagnoses. Moreover, we did not observe any breast cancer incidents during the follow-up period.

The management of IGM is controversial, and various treatment approaches have been used, ranging from observation to medical and surgical interventions [21]. Observational management may be suitable for cases where spontaneous healing occurs, which has been reported in approximately 50 % of patients. Medical treatment options for IGM include corticosteroids, Methotrexate, non-steroidal anti-inflammatory drugs, immunosuppressive agents like Azathioprine, antibiotics, and more recently, rifampin. Surgical treatments include wide local excision, incisional biopsy, drainage, and even mastectomy, depending on severity and individual patient factors. It is important to consider that individual patient characteristics, disease severity, and treatment protocols can all influence outcomes and recurrence rates in IGM.

The mainstay of treatment in the literature is wide local excision, either with or without corticosteroid therapy. Local excision has the shortest cure time but is associated with wound healing delays (10–50 %) and recurrence rates (8–38 %). Yukawa et al. reported a series of 13 IGM patients treated without corticosteroids, where 11 out of 13 patients required limited drainage of abscesses [22]. The time to resolution in their series ranged from 4 to 28 months. According to the retrospective study by Uysal et al. involving 720 IGM patients from multiple centers with various treatment approaches, >50 % of patients received a multimodal approach, and corticosteroid therapy was used in 39 % of patients, while 8 % underwent surgery alone [23]. The overall recurrence rate reported in their series was 17 %. According to the study findings by Shojaee et al. in 30 IGM patients, the recurrence rate with high-dose prednisolone treatment is 34.0 %, while it is around 17 % with low-dose prednisolone treatment [24]. In this study, the treatment approach did not involve corticosteroids or invasive procedures, and the recurrence rate of IGM within six months was lower than in similar studies that employed steroids alone or more invasive treatments. Additionally, our study showed a high healing rate with resolution of inflammation, pain, discharge, and fluctuation. These results suggest that cotrimoxazole may be a more favorable option than high-dose corticosteroids and a comparable alternative to low-dose corticosteroids regarding recurrence rates.

The exact mechanism by which cotrimoxazole exerts its effects in IGM is not fully understood, but some general considerations and potential mechanisms can be speculated. IGM is characterized histologically by the formation of non-caseating granulomas in the breast tissue, which suggests an inflammatory response. Although the exact cause of IGM remains unknown, it is believed to involve dysregulation of the immune system and an exaggerated inflammatory response. Cotrimoxazole can exert its effects through several mechanisms, including:

1. Antimicrobial Activity: Cotrimoxazole has broad-spectrum antimicrobial properties and is effective against a variety of bacteria, including both Gram-positive and Gram-negative organisms. While the role of infectious agents in the development or progression of IGM is not well established, some studies have reported the presence of bacteria in IGM specimens. Therefore, if bacterial infection plays a role in IGM pathogenesis, the antimicrobial activity of cotrimoxazole could help control or eliminate the infectious component, leading to symptom improvement [20]. In the present study, specifically according to presentations of patients, infectious origin is suspected in cases that may related to the successful response to the treatment by cotrimoxazole.

2. Anti-Inflammatory Effects: Cotrimoxazole, particularly sulfamethoxazole, has been shown to possess anti-inflammatory properties [20,25]. It may inhibit the production of inflammatory mediators, such as prostaglandins and leukotrienes, and modulate the immune response. By reducing inflammation in the breast tissue, cotrimoxazole could potentially alleviate the granulomatous inflammation associated with IGM and promote healing [27].

3. Immunomodulatory Effects: Cotrimoxazole has been reported to affect the immune system by modulating T-cell responses and altering cytokine production [28]. It may influence the balance between pro-inflammatory and anti-inflammatory cytokines, potentially leading to a more regulated immune response and decreased inflammation in IGM [28].

The limitations may be the number of cases in our study due to the rarity of IGM. Moreover, the study did not assess potential adverse effects or evaluate long-term recurrence rates beyond the 6 months. Considering these limitations, future studies with larger sample sizes and longer follow-up periods in randomized controlled trials are needed to confirm our findings and establish the optimal treatment protocol for IGM. Despite these limitations, our study provides valuable insights into the potential benefits of cotrimoxazole in treating IGM and highlights the need for further research in this area.

## 5 Conclusion

In conclusion, cotrimoxazole appears to be a promising treatment option for idiopathic granulomatous mastitis, with significant symptom reduction and a low recurrence rate observed in our study. Further research is needed to confirm these findings and to explore the long-term efficacy and safety of cotrimoxazole in the management of IGM.

## Informed consent

Informed consent was obtained from all individual participants included in the study.

## Ethical approval

Ethical approval was granted by the Ethics Committee of Shahid Beheshti University of Medical Sciences. Registration number IR.SBMU.MSP.REC.1402.076.

## Funding

This article did not receive fund.

## Author contribution

Dr. Majid Samsami, participated in Supervision and validation.

Dr. Fatemeh Parsaeian, participated in Conceptualization, Writing and Analysis.

Dr. Alireza Haghbin Toutounchi, participated in Methodology and Writing - Review & Editing, Analysis.

Dr. Hojatolah Khoshnoudi, participated in data curation and Visualization.

Dr. Hamed Tahmasbi, participated in Project administration, corresponding author.

## Guarantor

Dr. Majid Samsami accepts all responsibility of this article.

## Research registration number

N/A.

## Conflict of interest statement

All authors declare that they have no conflicts of interest.

## Data Availability

The datasets generated during and/or analyzed during the current study are available from the corresponding author on reasonable request.
